# Application of Galenic Strategies for Developing Gastro-Resistant Omeprazole Formulation for Pediatrics

**DOI:** 10.3390/children11080945

**Published:** 2024-08-05

**Authors:** Khadija Rouaz-El-Hajoui, Encarnación García-Montoya, Marc Suñé-Pou, Josep María Suñé-Negre, Pilar Pérez-Lozano

**Affiliations:** 1 Department of Pharmacy and Pharmaceutical Technology and Physical Chemistry, Faculty of Pharmacy and Food Sciences, University of Barcelona, Av. Joan XXIII, 27-31, 08028 Barcelona, Spain; khadijarouaz@ub.edu (K.R.-E.-H.); marcsune@ub.edu (M.S.-P.); jmsune@ub.edu (J.M.S.-N.); perezlo@ub.edu (P.P.-L.); 2 Pharmacotherapy, Pharmacogenetics and Pharmaceutical Technology Research Group, Bellvitge Biomedical Research Institute [IDIBELL], Av. Gran via de l’Hospitalet, 199-203, 08090 Barcelona, Spain

**Keywords:** omeprazole, pediatric pharmaceutical forms, gastro-resistance

## Abstract

Objectives: This study addresses a critical need in pediatric pharmacotherapy by focusing on the development of an enteric formulation of omeprazole for pediatric use. Omeprazole, a widely used proton pump inhibitor, is essential for treating various gastrointestinal disorders in children. The main objective is to design a compounding formula that can be prepared in hospital pharmacy services without the need for industrial equipment, which is often unavailable in these settings. Methods: The research applied different galenic strategies to overcome the challenges of omeprazole’s instability in acidic environments and its complex pharmacokinetic and physicochemical properties. The experiments were conducted sequentially, employing salting out, ionic gelation, and matrix granulation strategies. Based on the results obtained, the control conditions and parameters for the various trials were established. Results: Among the techniques used, wet granulation proved to be the most promising, achieving a gastro-resistance level of 44%. In contrast, the ionic gelation and salting-out techniques did not yield satisfactory results. Conclusions: The findings of this study underscore the need to adopt alternative formulation strategies to ensure the stability of omeprazole. This goal requires a multidisciplinary approach and continuous effort to design omeprazole formulations that meet quality standards and appropriate gastro-resistance requirements.

## 1. Introduction

Therapeutic orphanhood in the pediatric age group remains a reality, reflected in the scarcity of drugs adapted to the needs of pediatric patients. This challenge persists due to the lack of development of pharmaceutical forms specifically designed for this demographic group. Children present a constantly evolving biologic diversity, further complicating the search for effective and safe treatments. Despite this, there has been a growing interest in recent decades in the research and development of targeted drugs for both rare and more common pediatric diseases [1,2].

The lack of pediatric drugs available on the market directly impacts hospital pharmacy services, where compounding formulas are used to meet the needs of special patients, such as children, the elderly, or those with swallowing difficulties. In this context, drugs adapted for pediatric patients are developed, with extemporaneous oral liquid formulations being particularly suitable. These preparations allow doses to be precisely adjusted, either according to body weight or body surface area, ensuring safe and simple administration. Additionally, they avoid the complications associated with tablet or capsule ingestions in children [1,3,4].

Extemporaneous hospital preparations should be tested for quality and stability, as should marketed products. However, in practice, reliance is usually placed on available official information, such as that provided by the National Formulary, drug regulatory agencies, and specialized online literature. This is because hospital facilities lack the resources and means to conduct stability studies and comprehensive quality control. Consequently, there may be a lack of stability and quality studies, which can limit the use of many drugs in special patients [3].

Omeprazole is an active pharmaceutical ingredient (API) widely used in compounding formulas to treat gastrointestinal disorders in pediatric patients [5,6,7]. In most pediatric formulations, which are usually in suspension form, omeprazole is used without an enteric coating, which affects its effectiveness. The stability of this API is closely linked to pH: it decomposes rapidly in acidic environments, while it remains practically intact in alkaline media [8,9]. To counteract this acid degradation and favor its absorption in the duodenum, it is administered orally in capsules containing enteric pellets.

To develop a pediatric formulation that meets the gastro-resistance requirements necessary to maintain the effectiveness of omeprazole, several galenic technologies were investigated to microencapsulate it and avoid its exposure to the acidic environment of the stomach. It is essential to note that these techniques were chosen with the aim of developing a master formulation that can be produced without the need for complex industrial equipment. This decision is based on the conception that the formulation is designed for use in hospital pharmacy services.

### Theoretical Background: Formulation Strategies

Formulation strategies for pediatric use, particularly in hospital preparations, face significant challenges due to the necessity of customizing drugs for this population. These challenges encompass the stability of active ingredients, ease of administration, acceptance by children, personalized dosing, and safety and tolerability [10,11].

- The stability of active substances is critical since pediatric drugs must maintain chemical and physical stability over time. Active ingredients like omeprazole are especially sensitive to adverse conditions such as stomach acid pH, light, and heat. Therefore, formulations must shield these APIs from premature degradation to ensure therapeutic efficacy.
- Children, especially younger ones, may struggle with swallowing tablets or capsules. Formulations should be easily administered, whether through liquid forms, suspensions, dissolutions, or chewable tablets. Simplified administration facilitates adherence to treatment for caregivers.
- The taste and texture of drugs are crucial factors in ensuring acceptance by children. Formulations should mask bitter tastes and be palatable to prevent treatment rejection.
- Personalized dosing is essential due to variations in children’s weight and metabolism. Formulations should allow for flexible and precise dosing to meet the individual needs of each pediatric patient.
- Ensuring the safety and tolerability of formulations involves avoiding excipients that may cause allergies or adverse reactions, and minimizing side effects to ensure the treatment is well tolerated.

In hospital settings, extemporaneous preparations enable adjustment of doses and formulation of certain active ingredients not commercially available in pediatric-appropriate dosage forms. However, these preparations have limitations, including the reproducibility of formulations and the consistency needed to ensure safety [10,11].

The study employed several formulation strategies, starting with ionic gelation. This technique relies on ionic interactions between a polymer and a low-molecular-weight polyanion or polycation, resulting in an insoluble gel that encapsulates the API. This encapsulation protects the API within a polysaccharide matrix, with release occurring through gel phase changes [12,13]. Alginate, utilized for its release modulation, biodegradability, and safety properties, facilitates the gelation process by forming a hydrogel through an ionic reaction between COO- groups and divalent cations like calcium or strontium [14,15,16]. Ionic gelation is a straightforward, cost-effective method that operates under mild conditions, avoiding the need for high temperatures, vigorous agitation, or organic solvents, which could degrade sensitive active principles [12,13,14].

Another technique employed in the study was salting out. This method is advantageous for microsphere processing as it avoids the use of volatile organic solvents, such as organochlorine solvents. Instead, this technique uses a water-soluble organic solvent, like acetone, which is emulsified with an aqueous phase nearly saturated with salt. Although acetone is water-miscible, the high salt concentration in the aqueous phase prevents it from mixing with the organic phase [17]. In this process, the polymer and the API are dissolved in a water-soluble organic solvent like acetone, which is then emulsified with an aqueous phase saturated with salt. Stirring induces emulsion formation with an oily external phase (A/O), followed by phase inversion upon water addition, resulting in microsphere dispersion with an aqueous external phase (O/A) [17,18].

Finally, the study applied the wet granulation technique to produce enteric microspheres of omeprazole suitable for pediatric use. This process involves adding a binder dispersed in a liquid (typically water) over the substances to be granulated, forming a solution or suspension. The choice of binder liquid ensures that the substances do not dissolve, thereby forming a consistent granulate [19,20,21,22].

Inert matrices, commonly referred to as plastic or insoluble matrices, create solid, porous structures composed of non-toxic, non-digestible substances that remain intact in the gastrointestinal tract and are excreted in feces. Modern chemistry offers a wide range of excipients that form a porous structure which is resistant to gastrointestinal variations like pH, ionic concentration, enzymatic activity, or motility, except for drugs sensitive to pH changes [23,24]. Drug release from these matrices occurs through diffusion via pores, influenced by drug concentration, solubility, additives, granulation fluid properties, excipient particle size, and matrix system geometry. Liquids penetrate the porous network, enabling drug dissolution and diffusion through capillary-filled channels [23,24].

## 2. Materials and Methods

Omeprazole (CAS no. 73590-58-6) was kindly donated by Esteve Química (Barcelona, Spain).

Omeprazol base, sodium bicarbonate (CAS no. 144-55-8), sodium saccharin (CAS no. 128-44-9), ethyl cellulose N 100 (CAS no. 9004-57-3), polyvinylpyrrolidone (CAS no. 9003-39-8), decahydrate sodium tetraborate (CAS no. 1303-96-4) and polysorbate 80 (CAS no. 9005-65-6) were purchased from Fagron Ibérica SAU (Terrassa, Spain).

Calcium chloride dihydrate (CAS no. 10035-04-8), sodium alginate (CAS no. 9005-38-3), disodium phosphate (CAS no. 7558-79-4), monosodium phosphate (CAS no. 7558-80-7), sodium acetate (CAS no. 127-09-3), acetic acid (CAS no. 64-19-7), sodium chloride (CAS no. 7647-14-5), hydrochloric acid 5 M (CAS no. 7647-01-0), acetone (CAS no. 67-64-1), acetonitrile (CAS no. 75-05-8), and 96% ethanol (CAS no. 64-17-5) were purchased from PanReac Química S.L.U. (Barcelona, Spain).

Eudragit^®^ NE 30 D was kindly donated by Evonik Corp. (Barcelona, Spain).

The water used for analysis was Mili-Q grade. All solvents used were analytical grade. The HPLC column used in the analysis was a Nova Pack C-8 60 A 150 × 3.9 mm, with an internal diameter 4 mm.

### 2.1. Performance of 2 mg/mL Omeprazole Suspension in Xanthan Gum under Different pH Conditions

The behavior of the 2 mg/mL omeprazole suspension in xanthan gum (see Table 1) was studied in different pH media to simulate the pH variations in the stomach.

The suspension of omeprazole 2 mg/mL in xanthan gum was prepared according to the protocol provided by the pharmacy service of the Hospital Materno-Infantil Vall d’Hebron [25]. The gastro-resistance of the suspension at different pH levels was tested. To this end, three media with pH values of 1.2, 2.2, and 4.5 were prepared.

- The pH 1.2 medium was prepared by mixing 425 mL of 0.2 M HCl and 250 mL of NaCl 0.2 M and diluting the mixture to 1000 mL with Mili-Q water.
- The pH 2.2 medium was prepared by mixing 39 mL of 0.2 M HCl and 250 mL of NaCl 0.2 M and diluting the mixture to 1000 mL with Mili-Q water.
- The pH 4.5 medium was prepared by dissolving 2.99 g of sodium acetate in 250 mL of Mili-Q water, adding 14 mL of 2 M acetic acid, and diluting the mixture to 1000 mL with Mili-Q water.

Then, 10 mL of the suspension, equivalent to 20 mg of omeprazole, was added to each 900 mL of the respective pH media. The behavior of the suspension was observed over a period of 2 h.

Additionally, a placebo was prepared to verify that any color change in the pH media was not due to the degradation of any excipient. Three media with a pH of 1.2 were prepared: in the first, 10 mL of placebo was added; in the second, 20 mg of omeprazole enteric pellets was added; and in the third, 20 mg of omeprazole base was included. These media were also observed for 2 h. This approach aimed to demonstrate that the color changes observed in the pH media are due to the degradation of omeprazole in an acidic environment [26].

### 2.2. Obtaining Omeprazole Microspheres by Ionic Gelation

In this study, four experiments (see Table 2) were conducted using the ionic gelation technique to fabricate sodium alginate microspheres containing omeprazole. Alginate was chosen as the enteric polymer due to its extensive use in previous research and many applications, which have demonstrated its ability to modulate drug release, as well as its biodegradability and lack of toxicity. Alginate microspheres begin to release omeprazole when the pH of the environment exceeds 7, preventing its degradation in the stomach and allowing its release in the small intestine [16].

In the first experiment, 8.00 g of omeprazole was added to a hydroalcoholic solution composed of 80 mL of water and 4 mL of ethanol, along with 3.25 g of sodium alginate and 4.75 g of sodium bicarbonate. This mixture was stirred until a homogeneous dispersion was obtained. This high amount of omeprazole was specifically chosen to observe the behavior of the technique under high-API-load conditions. The dispersion was then poured using a hypodermic syringe into a second aqueous solution containing 5% [*w*/*w*] calcium chloride dihydrate. Microspheres formed immediately and were left in the original solution for 24 h to ensure internal gelation. They were then filtered, washed, and dried at room temperature.

In the second experiment, 1.50 g of sodium alginate was dispersed in 50 mL of purified water, under mechanical agitation until fully homogenized. The mixture was heated to 60 °C to facilitate the dispersion of sodium alginate in water. Simultaneously, 0.50 g of omeprazole was dissolved in a mixture of 12 mL of 96° ethanol and 8 mL of purified water. The omeprazole solution was kept under magnetic stirring until fully solubilized. It was then slowly added to the sodium alginate dispersion with continuous mechanical stirring to achieve a homogeneous mixture. Using a hypodermic syringe, the final mixture was added to a 15% [*w*/*w*] calcium chloride dihydrate solution, prepared beforehand. This last step was performed slowly to avoid aggregation and ensure uniform microsphere size. The microspheres formed were left in contact with the calcium chloride dihydrate solution for 30 min, then filtered, washed, resuspended in a 0.1% sodium alginate solution for 60 min, re-filtered, washed, and dried at room temperature for 24 h.

In the third experiment, the same method described in the second experiment was followed, but the amounts of sodium alginate and omeprazole were adjusted to 1.00 g each.

In the fourth experiment, experiments 2 and 3 were repeated, with the dispersions and solutions adjusted to a pH between 8 and 9 to minimize the degradation of omeprazole that occurs in acidic or slightly acidic media.

After drying, the microspheres were transferred into a 100 mL volumetric flask. Subsequently, 15 mL of ethanol was added, and the mixture was sonicated for 15 min. Then, 30 mL of 0.1 M sodium borate solution was added and sonicated again for 5 min. The mixture was allowed to equilibrate to room temperature, and the volume was adjusted to 100 mL with 0.1 M sodium borate solution. An aliquot was filtered, and the concentration of dissolved omeprazole was quantified using HPLC with UV–vis detection (Agilent Series 1100, Waldbronn, Germany).

### 2.3. Obtaining Omeprazole Microspheres by Salting Out

**Figure 1:**
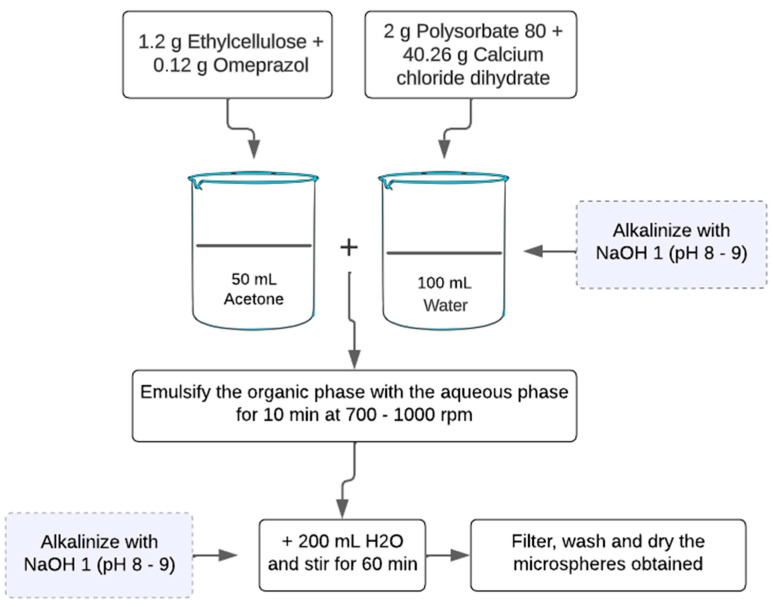
Schematic representation of the experiments conducted using the salting-out methodology. In Experiment 2, the aqueous solutions were alkalinized, in contrast to Experiment 1.

In this study, two experiments (see Figure 1) were carried out using the salting-out technique to fabricate omeprazole microspheres.

In the first experiment, 1.2 g of ethyl cellulose N 100 (a gastro-resistant polymer) was dissolved in 50.0 mL of acetone under magnetic stirring. Then, 0.12 g of omeprazole was added and stirred until completely dissolved. Meanwhile, 2.0 g of polysorbate 80 was added to 100 mL of purified water under magnetic stirring. Next, 40.26 g of calcium chloride dihydrate was added while maintaining mechanical stirring. The organic phase was added to the aqueous phase and emulsified for 10 min under mechanical stirring with paddles at 700–1000 rpm. Then, 200 mL of purified water was added, and the agitation was maintained for 1 h. Finally, the resulting microspheres were filtered, washed, and dried at room temperature.

In the second experiment, the methodology described in the first experiment was followed, but the aqueous phase and the water added at the end of the process were alkalinized (pH between 8 and 9) to prevent API degradation in acidic or slightly acidic pH media.

After the drying process, the microspheres were transferred to a 100 mL volumetric flask. Then, 15 mL of ethanol was added, and the mixture was sonicated for 15 min. Next, 30 mL of 0.1 M sodium borate solution was added and sonicated again for 5 min. The mixture was allowed to reach room temperature, and the volume was adjusted to 100 mL with 0.1 M sodium borate solution. An aliquot was filtered, and the amount of dissolved omeprazole was determined using HPLC with UV–vis detection (Agilent Series 1100, Waldbronn, Germany).

### 2.4. Obtaining Enteric Inert Matrices Using the Matrix Pelletizing Technique

In this study, three experiments were carried out using the matrix granulation technique to develop enteric inert matrices containing omeprazole. To impart gastro-resistance properties to the formulation, the excipients Eudragit^®^ NE 30D, ethyl cellulose N 100, and sodium alginate were selected (see Table 3).

In formula F1, 64.67 g of omeprazole was incorporated and mixed with 3.88 g of polyvinylpyrrolidone in a mortar until complete homogeneity was achieved. This mixture was then wetted with 31.65 g of Eudragit^®^ NE 30D dispersion (a liquid dispersion of neutral copolymers based on ethyl acrylate and methyl methacrylate). The resulting mixture was passed through a 2 mm sieve and dried for 24 h at 40 °C. Subsequently, the granulate was sieved again through a 1 mm sieve and stored in an amber container.

In formulations F2 and F3, 50 g of omeprazole was incorporated and mixed with 3 g of polyvinylpyrrolidone in a mortar until complete homogeneity was achieved. Then, 47 g of ethyl cellulose N 100 was added in F2, and 47 g of sodium alginate was added in F3. In both cases, kneading was carried out using water and 96° ethanol. The mixture obtained was passed through a 2 mm sieve and dried for 24 h at 40 °C. Subsequently, the granulate was sieved again through a 1 mm sieve and stored in an amber container.

Finally, a quantity equivalent to 20 mg of omeprazole for each granulate was weighed and added to a medium with a pH of 1.2, then stirred with paddles at 100 rpm for 1 h at a temperature of 37 ± 0.5 °C. After the test, the omeprazole pellets were recovered by filtering through a 0.2 mm sieve and washing with deionized water. The pellets were then transferred to a 100 mL volumetric flask, 15 mL of ethanol was added, and the mixture was sonicated for 15 min. Afterward, 30 mL of 0.1 M sodium borate solution was added and sonicated again for 5 min. The solution was allowed to cool and was made up to volume with 0.1 M sodium borate solution. An aliquot was filtered, and the amount of dissolved omeprazole was determined by UV–vis HPLC (Agilent 1100 Series, Waldbronn, Germany).

## 3. Results

### 3.1. Suspension of Omeprazole 2 mg/mL in Xanthan Gum in Different pH Media

**Figure 2:**
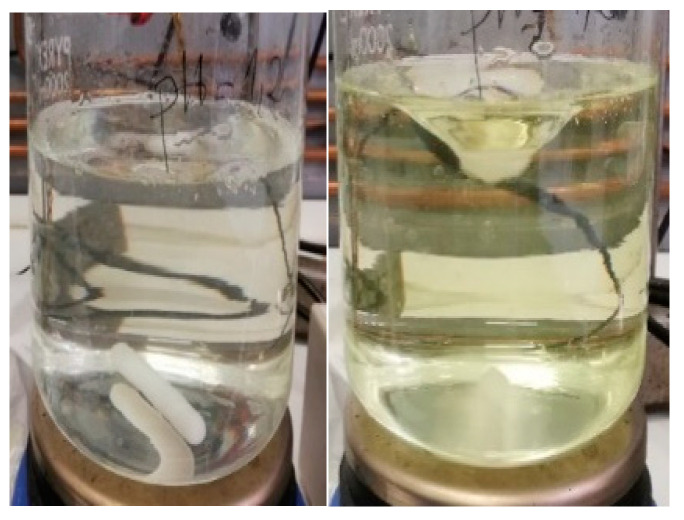
pH 1.2 medium. Left image: omeprazole 2 mg/mL suspension in xanthan gum at 0 min. Right image: omeprazole 2 mg/mL suspension in xanthan gum at 5 min.

As observed in Figure 2, when 10 mL of the 2 mg/mL omeprazole suspension in xanthan gum was added to the medium with a pH of 1.2, a color change from clear to slightly yellow was noted within 1 min. This coloration intensifies after 5 min, as shown in the right image of Figure 2.

**Figure 3:**
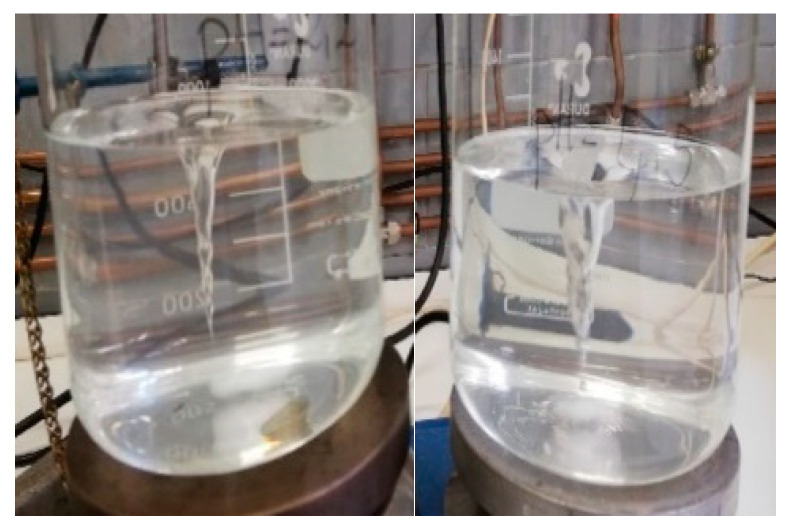
Left image: pH 2.2 medium; omeprazole 2 mg/mL suspension in xanthan gum at 5 min. Right image: pH 4.5 medium; omeprazole 2 mg/mL suspension in xanthan gum at 5 min.

In the pH 2.2 and 4.5 media (see Figure 3), after 5 min, they were no longer completely transparent, and a slight color change began to be observed.

**Figure 4:**
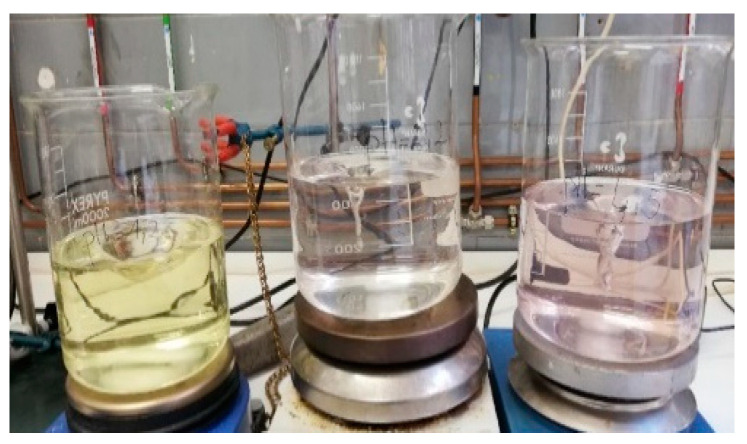
The three pH media after 1 h and 15 min. Left: pH 1.2 medium. Middle: pH 2.2 medium. Right: pH 4.5 medium.

**Figure 5:**
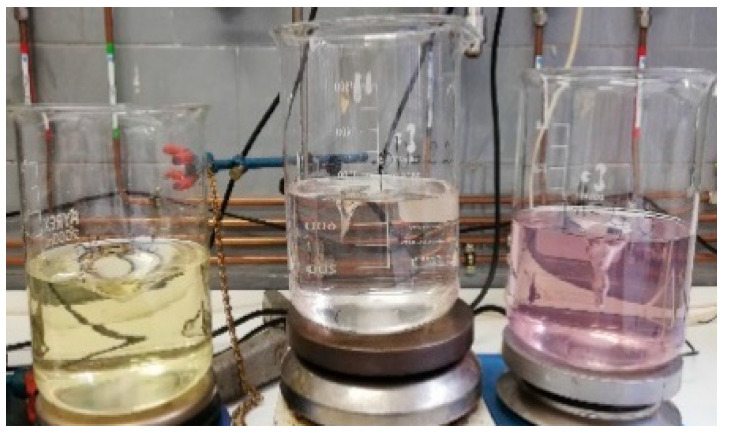
The three pH media after 2 h. Left: pH 1.2 medium (yellow color). Middle: pH 2.2 medium (light brown color). Right: pH 4.5 medium (pink color).

Figure 4 and Figure 5 show how the color changes given in each of the pH media intensify with time.

**Figure 6:**
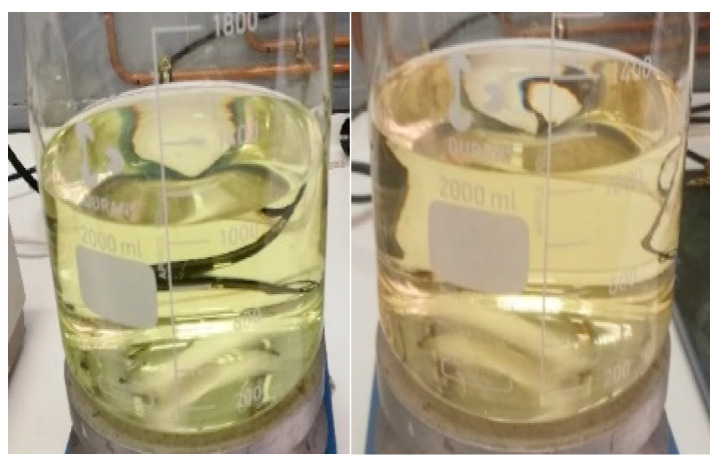
pH 1.2 medium with omeprazole base. Left image: 10 min after adding 20 mg of omeprazole base. Right image: 2 h after adding 20 mg of omeprazole base.

**Figure 7:**
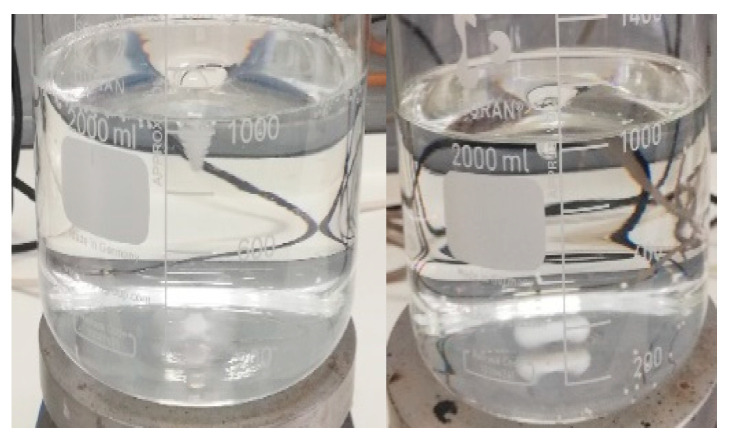
Left image: pH 1.2 medium with placebo. No color change was observed after 2 h. Right image: pH 1.2 medium with enteric-coated omeprazole pellets. No color change was observed after 2 h.

Figure 6 illustrates the behavior of omeprazole in an acidic medium (pH of 1.2): after 10 min, the medium turned completely yellow. In Figure 7 (left image), it is confirmed that the observed color changes are not attributable to the excipients used in the formulation. This is evidenced by adding 10 mL of placebo and observing its behavior in acid medium for 2 h, without any observed change in color. The same result was obtained when using omeprazole enteric pellets: no color change was detected, suggesting that the API does not undergo degradation in pH 1.2 medium (see Figure 7, image on the right).

### 3.2. Obtaining Omeprazole Microspheres by Ionic Gelation

**Figure 8:**
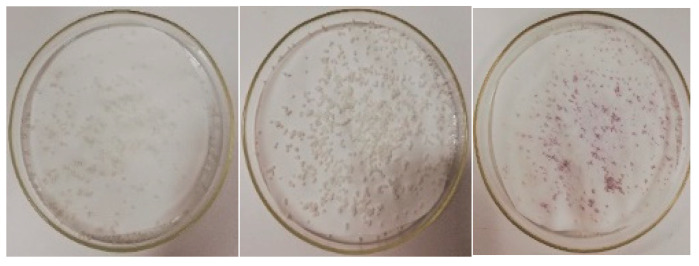
Left: microspheres from Experiment 2. Middle: microspheres from Experiment 3. Right: microspheres from Experiment 3 after the drying process.

**Figure 9:**
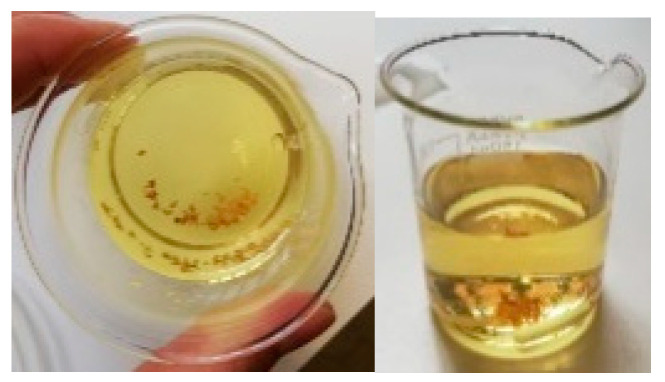
Left: microspheres from Experiment 2. Right: microspheres from Experiment 3 in acidic medium.

The microspheres resulting from Experiment 1 exhibited an irregular spherical appearance. After 5 min of exposure to 0.1 M hydrochloric acid medium, they acquired a yellowish-brown color; after 15 min, they begin to disintegrate; and after 30 min, they completely disintegrated, turning the medium yellow. Microspheres obtained in Experiments 2 and 3 displayed higher homogeneity compared to those of Experiment 1. However, their response to the acidic medium remained consistent across all experiments (see Figure 8 and Figure 9).

During the drying process, it was observed that the microspheres acquired a pinkish tone, indicating the degradation of omeprazole during the manufacturing process. In an attempt to prevent this degradation, Experiment 4 was carried out using dispersions and solutions with a pH between 8 and 9. However, it proved impossible to stabilize the formula, and similar results were observed: degradation of the API in hydrochloric acid medium and a color change in the microspheres after drying.

### 3.3. Obtaining Omeprazole Microspheres by Salting Out

**Figure 10:**
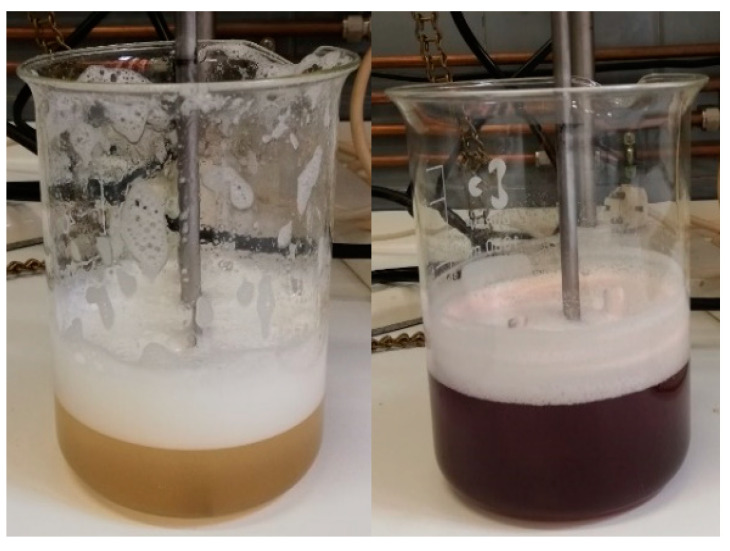
**Left**: appearance of the emulsion after 10 min of mechanical agitation with paddles. **Right**: appearance of the emulsion after 60 min of mechanical agitation with paddles.

**Figure 11:**
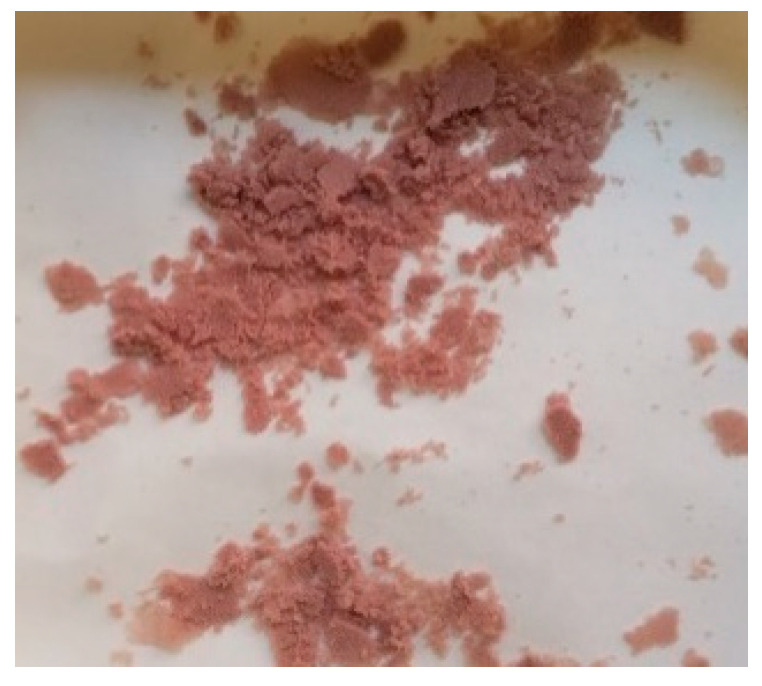
Appearance of the microspheres after the drying process.

In Experiment 1, the resulting emulsion acquired a yellow tone, which transformed into dark pink when 200 mL of purified water was added (see Figure 10). The microspheres obtained exhibited an intense pink color (see Figure 11). These color changes suggest the degradation of omeprazole during the microsphere elaboration process.

Therefore, in Experiment 2, the pH of the aqueous phase and of the water added at the end of the process was adjusted to a range between 8 and 9 to prevent this degradation. With this modification, the color changes observed previously in the emulsion did not occur. However, upon drying of the obtained microspheres, they still showed a pinkish hue, like that observed in Figure 11. It is worth mentioning that the residual acetone content was not determined because the microspheres obtained did not meet the gastro-resistance specifications.

### 3.4. Obtaining Enteric Inert Matrices Using the Matrix Granulation Technique

When using Eudragit^®^ NE 30 in F1 (Table 3), granules with a diameter of 100–200 µm were obtained. During the mixing process, a maximum amount of 31.65 g of Eudragit^®^ NE 30 in dry residue was added. It was not feasible to add a higher amount of this excipient, as doing so resulted in over-wetting of the kneading, hindering subsequent granulation. An attempt was made to mitigate this problem by wetting the mixture in successive phases to allow the addition of a higher quantity, but this strategy was not effective, since the final granulate acquired an excessively hard consistency, complicating its manual sieving. HPLC analysis of the granules obtained after a 1 h exposure to 0.1 M hydrochloric acid medium revealed a degradation of more than 56% of omeprazole.

In F2, when using an alcoholic solution of polyvinylpyrrolidone, the resulting granules disintegrated easily, preventing the formation of consistent matrices. When using an aqueous solution of polyvinylpyrrolidone, results similar to those of F1 were obtained. In both cases, the obtained minigranules showed a brown color change after 5 min of exposure to 0.1 M hydrochloric acid medium, indicating complete degradation of the API. Additionally, the medium also acquired a yellowish hue.

In F3, the use of aqueous polyvinylpyrrolidone solutions for the kneading process is not feasible, as it induces gelation of the sodium alginate. Therefore, it was decided that an alcoholic solution would be used, which produced results similar to those of F3: inconsistent minigranules were obtained, which easily fall apart. The behavior observed in acid medium was the same as in F2.

### 3.5. Analytical Results of Gastro-Resistant Test

**Figure 12:**
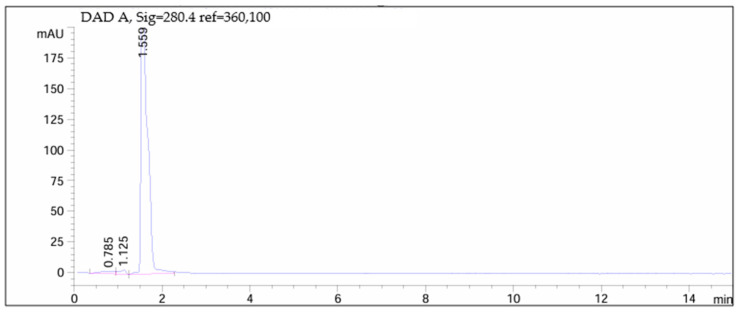
Chromatogram of omeprazole.

**Figure 13:**
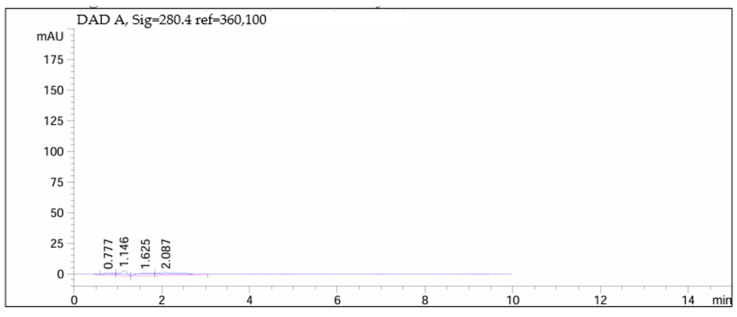
Chromatogram of omeprazole microspheres produced via ionic gelation.

**Figure 14:**
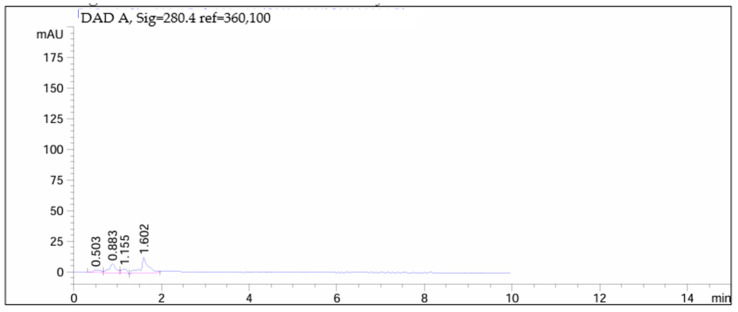
Chromatogram of omeprazole microspheres produced via salting out.

**Figure 15:**
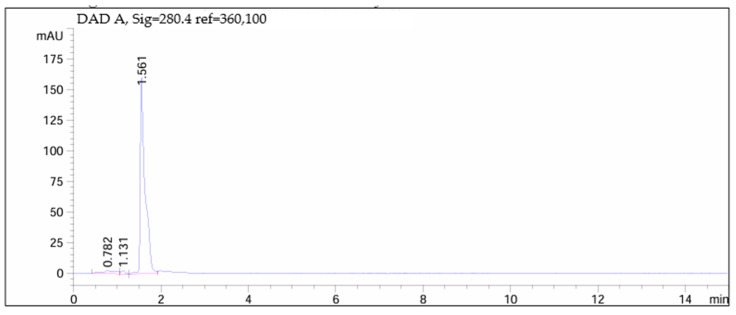
Chromatogram of omeprazole microspheres produced via wet granulation.

Figure 12, Figure 13, Figure 14 and Figure 15 depict chromatograms of the API and analyzed omeprazole microspheres obtained from each formulation strategy applied in the development process.

Figure 12 shows the chromatogram of the API, indicating a peak at 1.559 min.

Figure 13 and Figure 14 correspond to omeprazole microspheres obtained using the methods of ionic gelation and salting out, respectively. In both chromatograms, the peak corresponding to the API is absent due to degradation following exposure to 0.1 M hydrochloric acid for 2 h.

Figure 15 displays the chromatogram of omeprazole microspheres obtained from the wet granulation process after conducting the gastro-resistance test. Here, it is observed that the total API remains undegraded after exposure to 0.1 M hydrochloric acid for 1 h (API peak at 1.561 min).

## 4. Discussion

The stability of omeprazole as a raw material is closely linked to pH, being highly stable in alkaline solutions (pH greater than 7) but prone to degradation in acidic media. Consequently, omeprazole is typically marketed in capsule form with enteric pellets [9,27,28,29].

Compounding formulas of omeprazole, used to treat gastrointestinal disorders in pediatric patients, often face challenges in withstanding the acidic environment of the stomach and preserving the drug’s effectiveness. This is because suspensions incorporating omeprazole are generally used in alkaline mixtures. While these preparations may provide some protection to the API in an alkaline medium, they are insufficient to protect it from stomach acidity [30]. Moreover, the pharmacokinetic and chemical instability of omeprazole complicates its formulation in an aqueous medium [31,32].

When the compounding formulas of omeprazole come into contact with gastric acid, the protection they offer is compromised, resulting in premature degradation of omeprazole before it can reach its absorption site, the small intestine. The detailed study of the behavior of omeprazole 2 mg/mL suspension in xanthan gum in different acidic pH media provides a clearer understanding of this issue. It has been observed that when the suspension is added to acidic media, a color change occurs in the medium within a short time (less than 5 min). These color changes indicate omeprazole degradation in the gastric medium (see Figure 1, Figure 2, Figure 3, Figure 4, Figure 5 and Figure 6). Therefore, it is essential to consider alternative strategies to these preparations to ensure the necessary enteric protection when developing a pediatric compounding formula of omeprazole [33].

In the conducted study, various galenic techniques were employed to develop an enteric pediatric formulation of omeprazole. However, attempts to obtain enteric microspheres of omeprazole using sodium alginate via the ionic gelation method were also unsuccessful, as was the application of the salting-out technique. These results highlight the significant challenges presented by the omeprazole encapsulation process with the methodologies used thus far. On the other hand, when applying the matrix granulation technique using enteric excipients to confer gastro-resistance characteristics, better results were obtained with the use of Eudragit^®^ NE 30, although gastro-resistance levels higher than 44% were not achieved. It is worth mentioning that, generally, the use of gastro-resistant excipients such as Eudragit^®^ is not recommended in the pediatric population [1,4]. However, the pharmacokinetic characteristics and chemical instability of omeprazole in acidic environments justify their use. Thus, the strategies employed did not achieve the gastro-resistance levels required by the USP-NF [34], which specifies that after 2 h of exposure to a 0.1 M hydrochloric acid medium, no more than 15% of the API should be degraded.

It is worth mentioning that the selection of Eudragit NE and ethyl cellulose as enteric polymers instead of others (such as Eudragit L or S) is based on various technical and stability considerations. One of the main reasons for choosing Eudragit NE is that, in aqueous dispersion, this polymer has a pH of approximately 7, while Eudragit L and S have a slightly acidic pH, around 5. This acidic pH causes the degradation of omeprazole, an API sensitive to acidic conditions. Therefore, using Eudragit NE helps to prevent the degradation of omeprazole during the manufacturing process, ensuring the drug’s stability and efficacy. Furthermore, Eudragit NE is known for its ability to form microspheres that allow for controlled drug release in the gastrointestinal tract, offering a more predictable and consistent release profile [35]. On the other hand, ethyl cellulose is widely used as a film-forming and coating agent, providing modified release that can better meet the specific needs of the formulation [36].

The difficulties encountered in the development of a compounding formula of omeprazole, suitable for pediatric use and easy to prepare in hospital pharmacy services, are in line with what was stated by Ronchi F. and colleagues [37]. As they point out, the development of an oral liquid dosage form with modified release properties, aimed at improving adherence to treatment in patients with swallowing problems (such as pediatric or geriatric patients), is a challenge. This is because the modified-release dosage forms currently available on the market are mostly solid as capsules or tablets.

Finally, the results of this study developed by our research group underscore the complexity of developing a compounding formula without resorting to industrial-scale galenic formulation techniques, such as fluid-bed coating of inert pellets or the use of 3D drug printing methods. On the one hand, a previous study titled “Optimisation of the Manufacturing Process of Organic-Solvent-Free Omeprazole Enteric Pellets for the Paediatric Population: Full Factorial Design” [38] details the production of omeprazole enteric pellets with a diameter of 0.5–0.6 mm by fluid-bed coating. These pellets demonstrated optimal gastro-resistance and release properties, and due to their morphological characteristics, they are suitable for use in pediatric liquid preparations. Additionally, their small diameter facilitates their use in the 3D printing technique using semisolid extrusion, enabling uniform mixing with the carrier hydrogel and ensuring homogeneous distribution of the API. The pellet size optimizes the extrusion process by allowing controlled and precise flow, thereby improving the accuracy and resolution of printed formulations. This 3D technique is particularly beneficial for thermolabile APIs like omeprazole, as it minimizes exposure to high temperatures during manufacturing, reducing the risk of drug degradation [39]. These studies suggest that a possible solution to address the lack of gastro-resistance in most pediatric omeprazole compounding formulas is the development of enteric pellets with a suitable morphology to avoid swallowing problems. This should be carried out in drug development centers that comply with good manufacturing practice (GMP) standards, and these centers should supply the pellets so that hospital pharmacy services can use them in liquid preparations. If 3D printing techniques are used, they must also be GMP-compliant. In this way, formulations that meet quality standards and are acceptable and attractive to pediatric patients can be obtained.

**Figure 16:**
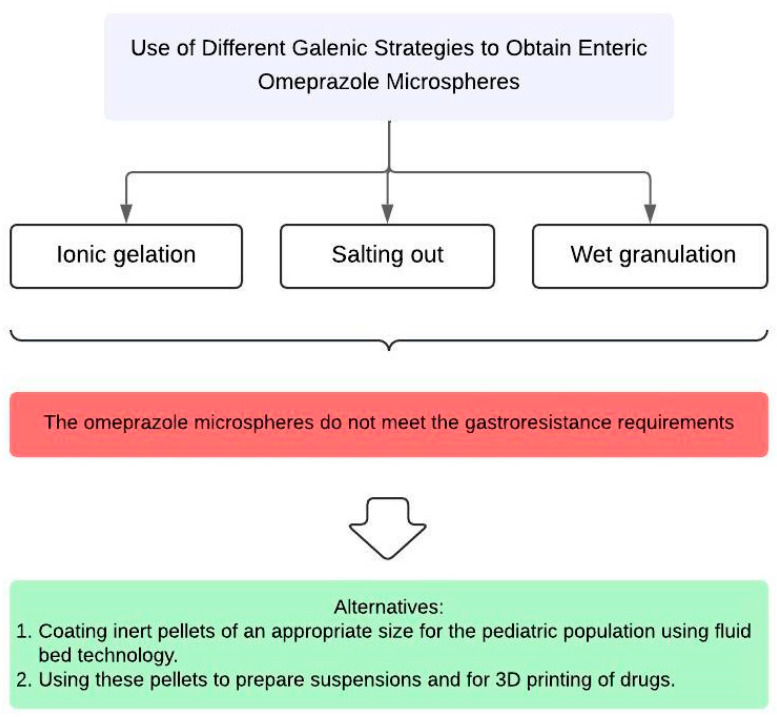
Schematic representation of the results obtained, and the possible alternatives proposed for obtaining gastro-resistant microspheres of omeprazole.

Figure 16 depicts a schematic representation of the results obtained and the possible alternatives proposed for obtaining gastro-resistant microspheres of omeprazole.

## 5. Conclusions

The present study highlights significant challenges in the development of enteric pediatric formulations of omeprazole, as the stability of this API is strictly influenced by several factors, with pH being the most critical. The evaluation of the behavior of omeprazole 2 mg/mL suspension in xanthan gum in different acidic pH media, commonly used in the treatment of gastrointestinal disorders in pediatric patients, reveals the lack of pediatric preparations with gastro-resistant properties necessary to ensure the effectiveness of this drug. Several techniques were employed in the study to develop enteric microspheres of omeprazole, including ionic gelation, salting out, and wet granulation, without the use of industrial equipment typically unavailable in hospital pharmacy services. Among these techniques, wet granulation proved to be the most promising, achieving a gastro-resistance level of 44%. In contrast, ionic gelation and salting-out techniques did not yield satisfactory results. It is important to note that the findings of this study underscore the need to adopt alternative formulation strategies to ensure the stability of omeprazole. This goal requires a multidisciplinary approach and continuous effort to design pediatric omeprazole formulations that meet quality standards and appropriate gastro-resistance requirements.

## Figures and Tables

**Table 1:** Composition of 2 mg/mL omeprazole suspension in xanthan gum [25].

Components	Percentage [%]
Omeprazole base	0.2
Sodium bicarbonate	8.4
Xanthan gum [aqueous solution 1%]	50 mL
Vanilla essence	0.1–0.2
Sodium saccharin	0.1–0.3
Purified water q.s.	100 mL

**Table 2:** Scheme of the experiments carried out following the ionic gelation methodology.

Critical Parameters for Each Experiment	Experiment 1	Experiment 2	Experiment 3	Experiment 4
Omeprazole amount [g]	8.00	0.50	1.00	0.50–1.00
Sodium alginate amount [g]	3.25	1.50	1.00	0.50–1.00
Aqueous solution of calcium chloride dihydrate	5% [*w*/*w*]	15% [*w*/*w*]	15% [*w*/*w*]	15% [*w*/*w*]
Resuspension in 0.10% sodium alginate solution for 24 h	No	Yes	Yes	Yes
Alkalinization of solutions and dispersions used	Use of sodium bicarbonate, pH between 7 and 8 [4.75% [*w*/*w*]]	pH adjustment to 8–9 with NaOH 1 N	pH adjustment to 8–9 with NaOH 1 N	pH adjustment to 8–9 with NaOH 1 N

**Table 3:** Composition of the experiments conducted using the matrix granulation methodology.

Components	F1	F2	F3
Omeprazole	64.67 g	50.00 g	50.00 g
Eudragit^®^ NE 30 D	31.65 g [dry residue]	---	---
Ethyl cellulose N 100	---	47.00 g	---
Sodium alginate	---	---	47.00 g
Polyvinylpyrrolidone	3.88 g	3.00 g	3.00 g

## Data Availability

The original contributions presented in the study are included in the article, further inquiries can be directed to the corresponding author.
